# Adaptive Scheme for Detecting Induction Motor Incipient Broken Bar Faults at Various Load and Inertia Conditions

**DOI:** 10.3390/s22010365

**Published:** 2022-01-04

**Authors:** Mohamed Esam El-Dine Atta, Doaa Khalil Ibrahim, Mahmoud Gilany, Ahmed F. Zobaa

**Affiliations:** 1 Department of Electrical Power Engineering, Faculty of Engineering, Cairo University, Giza 12613, Egypt; eng.mesam2010@gmail.com (M.E.E.-D.A.); doaakhalil73@eng.cu.edu.eg (D.K.I.); drgilany@eng.cu.edu.eg (M.G.); 2 Electronic and Electrical Engineering Department, College of Engineering, Design and Physical Sciences, Brunel University London, London UB8 3PH, UK

**Keywords:** broken bar faults (BBFs), Fast Fourier Transform (FFT), incipient BBFs, variable inertia, variable load, non-adjacent BBFs

## Abstract

This paper introduces a novel online adaptive protection scheme to detect and diagnose broken bar faults (BBFs) in induction motors during steady-state conditions based on an analytical approach. The proposed scheme can detect precisely adjacent and non-adjacent BBFs in their incipient phases under different inertia, variable loading conditions, and noisy environments. The main idea of the proposed scheme is monitoring the variation in the phase angle of the main sideband frequency components by applying Fast Fourier Transform to only one phase of the stator current. The scheme does not need any predetermined settings but only one of the stator current signals during the commissioning phase. The threshold value is calculated adaptively to discriminate between healthy and faulty cases. Besides, an index is proposed to designate the fault severity. The performance of this scheme is verified using two simulated motors with different designs by applying the finite element method in addition to a real experimental dataset. The results show that the proposed scheme can effectively detect half, one, two, or three broken bars in adjacent/non-adjacent versions and also estimate their severity under different operating conditions and in a noisy environment, with accuracy reaching 100% independently from motor parameters.

## 1. Introduction

Induction motors are the backbone of many industries. Detection of motor faults in their incipient phases reduces consequential and extended damage, hence decreasing repairing cost and unscheduled shutdown and its related cost [1].

Motor faults arise from defects in bearing, stator, rotor, and other causes. The detection of cage faults is discussed in this paper. This fault can be detected in both starting and steady-state conditions. Many papers discussed the detection of broken bar faults (BBFs) under starting conditions such as [1,2,3]. However, in the real world, the motor can run continuously for several hours without starting in many applications. Therefore, in this paper, we will concentrate on the detection of such fault under steady-state conditions.

The frequency spectrum analysis of motor current using Fast Fourier Transform (FFT) is the most common method for detecting BBFs and some other electromechanical faults [4,5]. The broken bar fault (BBF) increases the magnitude of frequency components of the order equal to$\;\left[\left(1\pm 2ks\right)f\right]$, where k is an integer starting from 1, s is the motor slip and f is the supply frequency. At k = 1, the frequency components are called the main sideband. These frequencies already exist in healthy conditions due to inherent asymmetry [4]. These frequency components are sensitive to system inertia as discussed in [6,7]. However, this effect has not been deeply investigated in many research studies [8,9,10]. The popularity of this method is because of its non-invasive nature and because it only requires the measurement of a stator current through current sensors [4,11]. Conversely, it has many drawbacks such as spectral leakage, non-suitability for variable loading, and the difficulty in calculating the threshold that is usually determined based on the experience that makes some faults undetectable [5,7,11].

Several ideas were reported in the literature to solve these problems. Wavelet transform was applied in [10]; however, it is greatly dependent on the selection of the mother wavelet and not suitable for light loads [10,11]. Hilbert–Huang transform (HHT) as well as Empirical mode decomposition (EMD) were used to diagnose BBFs and indicated a significant improvement in Motor Current Signature Analysis (MCSA), especially at low slip and detection of incipient faults [11]. However, they are sensitive to stopping criteria and an end-point approach [11]. Moreover, the Hilbert transform was used in [12] for current envelope extraction to diagnose BBFs. Park’s transform was also used to detect BBFs, but the main disadvantage of this method is that it cannot differentiate between the broken bar and other faults. Dependence on the number of poles and slot number and the three-phase current signals were required [13,14]. Novel modifications were applied to that method to overwhelm its dependence on the number of poles and slot numbers in [14].

Later, high-resolution techniques such as MUSIC (MUltiple SIgnal Classification) and ESPRIT (Estimation of Signal Parameters via Rotational Invariance Techniques) were used [11,13,15,16]. However, they are sensitive to sensor accuracy and position; furthermore, these methods are time-consuming, as they need a high computational burden [11]. Besides, it was claimed in [13,17] that ESPRIT suffers from an accuracy problem in amplitude and phase estimation [11,13]. In an attempt to overcome the complexity and the need for a high computational burden of these techniques, a method based on the theory of Rayleigh quotient was proposed in [18].

Further research studies are currently conducted toward combining artificial intelligence (AI) with the aforementioned methods to solve the threshold determination challenges and classification problems, as in [8,19,20,21,22]; nevertheless, large training data are required. Another attempt to overcome the difficulties for the threshold estimation was the proposed scheme in [23] to detect and classify BBFs with no setting. However, the detection and classification procedures were based on the experience and the effect of inertia variation was not studied in such a scheme. 

It is noteworthy that the detection of non-adjacent 2 broken bars (BBR) fault and half-broken bar (HBBR) is still a challenge. A flux sensor was proposed in [24,25] to detect non-adjacent 2 BBR fault, while the stator zero-sequence current that requires three-phase current sensors was analyzed in [26] to detect the same fault. Besides, the HBBR can be detected using the MUSIC technique in [27], but it requires a long acquisition time and complicated threshold calculations; therefore, a modification was proposed in [28] to limit the drawbacks of such technique. The vibration signal was also used for detecting incipient BBR in [29] using AI-based technique, while one-phase current signal and neural network were used for HBBR fault detection in [30].

Therefore, the novelty of this article can be summarized in the following:

▪ Proposing a novel scheme based on an analytical approach to detect and diagnose BBFs. Hence, the results are easy to interpret. Furthermore, no training process is required.
▪ The proposed scheme does not need any settings instead; it employs an adaptive threshold to discriminate between healthy and faulty cases under different operating conditions.
▪ The proposed scheme can effectively detect incipient BBFs and non-adjacent BBFs, representing a stumbling block to many other methods in the literature.
▪ The proposed scheme can precisely detect BBFs under variable loading and different inertia conditions.
▪ The proposed scheme is immune to high-level noise and independent from motor parameters.

The organization of this paper is presented as follows: the simulation of an induction motor with BBF is described in Section 2. The methodology of the proposed scheme relying on the phase angles of main sideband components is offered in detail in Section 3, whereas the effect of inertia variation, load variation, and BBFs are investigated in Section 4. The detailed stages to implement the proposed scheme are presented in Section 5, including the data acquisition stage, data processing stage, adaptive threshold determination, fault detection, and severity index calculation. Investigating the performance of the proposed scheme using two simulated motors with different designs in addition to a real experimental dataset is extensively discussed in Section 6. Finally, the conclusions are drawn in Section 7.

## 2. Simulating Broken Bar Faults in Induction Motor

The finite element method (FEM) is used to simulate two tested motors of the same power but with different parameters as listed in Table 1. The data for Motor I is obtained from [3], while Motor II is found in ANSYS Maxwell 16.0 RMxprt example library and is named “yz200-6”.

Motor I has been simulated extensively at different fault severity levels under variable loading conditions and with different load inertia whereas Motor II has been simulated for a limited number of cases to confirm that the proposed scheme can detect BBFs efficiently regardless of motor characteristics and design. 

FEM is selected for its high accuracy and the ability to model magnetic characteristics of silicon steel, the exact geometry of the motor, the stator winding, and rotor bar distribution [4]. ANSYS Maxwell software is applied to simulate motors models.

For Motor I, the healthy case model is simulated; then, models for a partial broken bar, one broken bar, two adjacent broken bars, two non-adjacent broken bars at one pole-pitch distance, and three adjacent broken bars are simulated. For further verification of the results, the model of Motor II at a healthy case and with a partial broken bar is simulated. 

**Figure 1:**
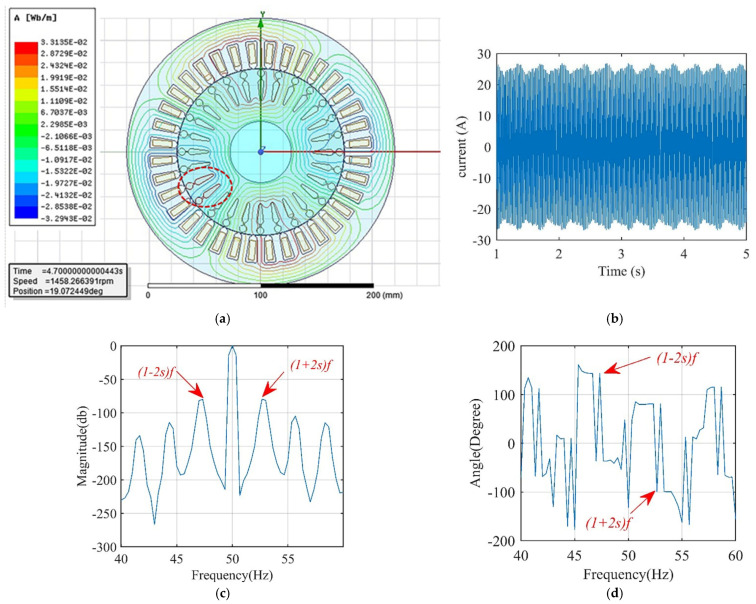
Characteristics for modeling Motor I with 2 BBR. (**a**) The magnetic flux lines distribution. (**b**) Periodic oscillation in the stator current envelop. (**c**) The main sideband frequency components for stator current (40–60 Hz). (**d**) Phase angle of the main sideband components.

A sample of the output data of Motor I with two adjacent broken bars (2 BBR) is illustrated in Figure 1. Figure 1a demonstrates the cross-sectional magnetic flux lines distribution with the two adjacent broken bars colored in red. The effect of BBF on the stator current in the time domain appears as a periodic oscillation in the current envelop as revealed in Figure 1b. The frequency spectrum of the stator current with the main sideband frequency components $\left[\left(1\pm 2ks\right)f\right]$ is displayed in Figure 1c. Finally, the phase angle of the main sideband components calculated using FFT is also shown in Figure 1d.

## 3. Methodology of the Proposed Scheme

One of the main objectives of this section and the next section is to draw a clear relationship between the magnitude and phase angle of the left and right main sideband components (refer to Figure 1c) in case of inertia variation, load variation, and broken bar fault occurrence. These cases are investigated graphically, and the main sideband components are derived mathematically by Equations (9) and (10). 

The other objective is to deduce a methodology for determining adaptively the threshold of the proposed scheme. It will be calculated using Equation (15) upon solving Equations (12) and (14). Then, to evaluate the motor status, the phase angle change will be compared against the estimated threshold. Finally, the severity index is calculated using Equation (24) and will be discussed in detail in Section 5.4.

It is well known that a backward field arises from rotor bar asymmetry with a frequency equal to sf that interacts with the stator magnetic field component, producing a current component $i_{1}^{ο}$ with frequency $\left[\left(1-2s\right)f\right]$ in the stator current, which has an instantaneous value that can be expressed by [4,31]:

(1) $$i_{1}^{ο}=\sqrt{2}I_{1}^{ο}sin\left[\left(1-2s\right)\omega t-\alpha_{I_{1}}\right]$$

where s denotes the motor slip and $\alpha_{I_{1}}$ is an angle relies on the transient state required to reach the average speed and on the rotor asymmetry position [31]. 

This current $\;i_{1}^{ο}$ besides the stator fundamental current interact with the fundamental flux linkage producing the motor average torque needed to drive its load and torque oscillations at different frequencies. The instantaneous value of the fundamental flux ($\lambda_{fund}$) can be expressed by the following equation, where  λ  denotes the root mean square (RMS) value of the fundamental flux linkage and $\alpha_{\lambda }$ is its angle [4].

(2) $$\lambda_{fund}=\sqrt{2}\;\lambda sin\left[\omega t-\alpha_{\lambda }\right]$$

These oscillations are filtered out by the system inertia and thus only torque oscillations at frequency $2sf_{s}$ remain which produce speed oscillations at the same frequency with different amplitude [4,31]. These speed oscillations induce two electromotive forces (EMFs) in the stator windings, described by the following equations [4]:

(3) $$e_{1-2s}=E_{1-2s}\cdot cos\left[\left(1-2s\right)\omega t-\alpha_{I_{1}}\right]$$

(4) $$e_{1+2s}=-E_{1+2s}\cdot cos\left[\left(1+2s\right)\omega t-2\alpha_{\lambda }+\alpha_{I_{1}}\right]$$

As discussed in [31], for the sake of simplicity, it is assumed that $E_{1-2s}≅E_{1+2s}≅E_{1}$ and the impedance $Z_{1-2s}≅Z_{1+2s}≅Z\;∠\phi$. Using the equations derived in [4] and knowing that the parameter $\beta =\frac{3P\lambda^{2}}{8Js\omega \left|Z\right|}$ where P,J and ω describe the number of poles, system inertia, and synchronous speed respectively, consequently, the two EMFs will produce the following currents:

(5) $$i_{1}^{\prime }=\frac{E_{1}}{\left|Z\right|}cos\left[\left(1-2s\right)\omega t-\alpha_{I_{1}}-\phi \right]\;\;\;\;\;\;\;\;\;\;\;\;\;\;\;\;\;\;=\frac{3P\lambda^{2}}{8Js\omega_{s}\left|Z\right|}I_{1}^{ο}cos\left[\left(1-2s\right)\omega t-\alpha_{I_{1}}-\phi \right]\;\;\;\;\;\;\;\;\;\;\;\;\;\;\;\;\;\;=\beta I_{1}^{ο}sin\left[\left(1-2s\right)\omega t-\alpha_{I_{1}}-\phi +\frac{\pi }{2}\right]\;\;\;\;\;\;\;\;$$

(6) $$i_{2}=\frac{-E_{1}}{\left|Z\right|}cos\left[\left(1+2s\right)\omega t-2\alpha_{\lambda }+\alpha_{I_{1}}-\phi \right]\;=\beta I_{1}^{ο}sin\left[\left(1+2s\right)\omega t-2\alpha_{\lambda }+\alpha_{I_{1}}-\phi +\frac{\pi }{2}\right]$$

where Z and ϕ  present the magnitude and the angle of the equivalent circuit impedance at supply frequency. 

Similar to the sequence for deducing $\;i_{1}$, the current $i_{2}$ induces a new magnetic flux component that interacts with the fundamental flux $\lambda_{fund}$ producing torque oscillations and speed oscillations at frequency equals $\;2sf_{s}$. These speed oscillations induce two EMFs in the stator windings that generate two current components described by the following equations:

(7) $$i_{1}^{\prime \prime \prime }=\beta^{2}I_{1}^{ο}sin\left[\left(1-2s\right)\omega t-\alpha_{I_{1}}\right]$$

(8) $$i_{2}^{\prime }=\beta^{2}I_{1}^{ο}sin\left[\left(1+2s\right)\omega t-2\alpha_{\lambda }+\alpha_{I_{1}}-2\phi \right]$$

Note that the currents $\;i_{1}^{\prime \prime \prime }\;and\;i_{1}^{ο}\;$ are in phase; thus, their resultant current $\;i_{1}\;$ is the algebraic summation of the two currents, so: $\;i_{1}=i_{1}^{\prime \prime \prime }+i_{1}^{ο}$.

The current $i_{1\;}$ interacts with the current $i_{1}^{\prime }$ producing a resultant current $\;i_{1}^{\prime \prime }$ (vector summation of $i_{1}$ & $i_{1}^{\prime }$) at frequency $\left[\left(1-2s\right)f_{s}\right]$, whereas the current $i_{2}^{\prime }\;$ interacts with the current $i_{2}$ producing a resultant current $i_{2}^{\prime \prime }$ at frequency $\left[\left(1+2s\right)f_{s}\right]$ equals to the vector summation of $i_{2}$ & $i_{2}^{\prime }$.

(9) $$\vec{i_{1}^{\prime \prime }}=\vec{i_{1}^{\prime }}+\vec{\;i_{1}^{\prime \prime \prime }}+\vec{i_{1}^{ο}}$$

(10) $$\vec{i_{2}^{\prime \prime }}=\vec{i_{2}}+\vec{i_{2}^{\prime }}$$

Accordingly, the RMS value of $i_{1}^{\prime \prime }$ can be expressed by:

(11) $$I_{1}^{\prime \prime }=\;\;\;\;\;\sqrt{{\left(\left(1+\beta^{2})I_{1}^{ο}cos\left(\theta_{1}\right)+\beta I_{1}^{ο}cos(\theta_{1}^{\prime }\right)\right)}^{2}+{\left(\left(1+\beta^{2})I_{1}^{ο}sin\left(\theta_{1}\right)+\beta I_{1}^{ο}sin(\theta_{1}^{\prime }\right)\right)}^{2}}$$

After some mathematical manipulations, it can be deduced that:

(12) $$I_{1}^{\prime \prime }{}^{2}={\left(1+\beta^{2}\right)}^{2}I_{1}^{ο}{}^{2}+2\beta \left(1+\beta^{2}\right)I_{1}^{ο}{}^{2}cos(\theta_{1}^{\prime }-\theta_{1})+\beta^{2}I_{1}^{ο}{}^{2}\;I_{1}^{\prime \prime }{}^{2}={\left(1+\beta^{2}\right)}^{2}I_{1}^{ο}{}^{2}+2\beta \left(1+\beta^{2}\right)I_{1}^{ο}{}^{2}cos(\frac{\pi }{2}+\phi )+\beta^{2}I_{1}^{ο}{}^{2}$$

whereas the RMS value of $i_{2}^{\prime \prime }$ can be expressed by:

(13) $$I_{2}^{\prime \prime }=\;\;\;\;\;\sqrt{{\left(\beta I_{1}^{ο}cos\left(\theta_{2}\right)+\beta^{2}I_{1}^{ο}cos(\theta_{2}^{\prime })\right)}^{2}+{\left(\beta I_{1}^{ο}sin\left(\theta_{2}\right)+\beta^{2}I_{1}^{ο}sin(\theta_{2}^{\prime })\right)}^{2}}$$

After some mathematical manipulations, it can be deduced that:

(14) $$I_{2}^{\prime \prime }{}^{2}=\beta^{2}I_{1}^{ο}{}^{2}+2\beta^{3}I_{1}^{ο}{}^{2}cos(\theta_{2}^{\prime }-\theta_{2})+\beta^{4}I_{1}^{ο}{}^{2}\;I_{2}^{\prime \prime }{}^{2}=\beta^{2}I_{1}^{ο}{}^{2}+2\beta^{3}I_{1}^{ο}{}^{2}cos(\frac{\pi }{2}+\phi )+\beta^{4}I_{1}^{ο}{}^{2}$$

The proposed scheme is based on monitoring $\;\theta_{1}^{\prime \prime }$, $\theta_{2}^{\prime \prime }$ variations due to the changes in load inertia, motor loading, and BBFs occurrence that will be discussed and investigated graphically in the next section supported with simulated cases of the two motor models. However, to draw a quick conclusion of these effects on $\;\theta_{1}^{\prime \prime }$, $\theta_{2}^{\prime \prime }$ variations to continue this section, it can be mentioned that the parameter β changes with any inertia change, loading condition variation, or BBFs occurrence whereas the parameter $i_{1}^{ο}$ is dependent on loading conditions and motor asymmetry degree. Consequently, the magnitudes of $i_{1}^{\prime },\;i_{2},\;i_{1}^{\prime \prime \prime }and\;i_{2}^{\prime }$ differ with the variation in the aforementioned conditions and hence the angles of the main sideband $\;\theta_{1}^{\prime \prime }$, $\theta_{2}^{\prime \prime }$ will vary, as described in Equations (9) and (10). 

These angles of the main sideband $\;\theta_{1}^{\prime \prime }$, $\theta_{2}^{\prime \prime }$ can be changed due to their internal variation regardless of the changes in β and $i_{1}^{ο}\;$ such as $\alpha_{\lambda }$ changes in case of BBF occurrence and ϕ changes in case of load variations. However, as a general rule, for healthy conditions only, the change due to internal phase variation in the phase angle of the left and right main side band components is equal but at different sign $∆\theta_{1}^{\prime \prime }=-∆\theta_{2}^{\prime \prime }$ after the compensation of ϕ variation.

Therefore, to calculate $\;∆\theta_{1}^{\prime \prime },\;∆\theta_{2}^{\prime \prime }$, a reference case is required, which may be any healthy case with any load inertia and under any loading condition. Using the magnitudes of $i_{1r}^{\prime \prime }\;and$ $i_{2r}^{\prime \prime }$ calculated from FFT of the reference case (r) stator current, Equations (12) and (14) are solved. Accordingly, the values of $\beta_{r}$ and $I_{1r}^{ο}$ for the reference case are obtained. Similarly, the values of $\beta_{new}$ and $I_{1new}^{ο}$ for any new case (new) can be calculated. These two parameters are the keys to determine the magnitudes of $i_{1}^{\prime },\;i_{2},\;i_{1}^{\prime \prime \prime }and\;i_{2}^{\prime }\;$ using Equations (5)–(8). 

**Figure 2:**
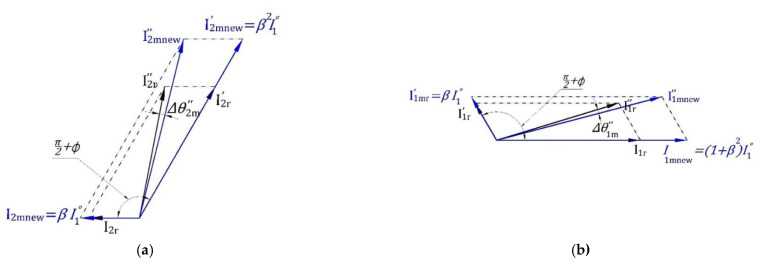
The effect of main sideband current components magnitude variation on their angles variation. (**a**) The effect of right sideband component magnitude variation on $\;∆\theta_{2}^{\prime \prime }$. (**b**) The effect of left sideband component magnitude variation on $∆\theta_{1}^{\prime \prime }$.

Now, we should discriminate between the variation in angles $\;\theta_{1}^{\prime \prime }$, $\theta_{2}^{\prime \prime }$ resulting from the magnitude variation and that from the internal angles variations resulting from fault occurrence. For this purpose, the model in Figure 2 is constructed to study the effect of magnitude variation (β and $I_{1}^{ο}$ variation) on $∆\theta_{1}^{\prime \prime },\;∆\theta_{2}^{\prime \prime }$ where the black lines represent the normal reference case (with the subscript r), and the blue lines represent any new case (with the subscript new), while the subscript (mnew) indicates the new case current components calculated using the model in Figure 2.

Using this model, the phase angle deviation of left and the right main sideband components from the reference case due to magnitude variation $\Delta \theta_{1m}$ and $\Delta \theta_{2m}$ (*m* denotes to model) can be determined, and hence the adaptive threshold value can be estimated as follows:

(15) $$\Delta \theta_{1m}=\theta_{1r}^{\prime \prime }-\theta_{1\mathrm{mnew}}^{\prime \prime }\;\Delta \varphi =\varphi_{r}-\varphi_{new}\;\Delta \theta_{2m}=\theta_{2r}^{\prime \prime }-\theta_{2\mathrm{mnew}}^{\prime \prime }+\Delta \varphi \;threshold=\Delta \theta_{1m}+\Delta \theta_{2m}$$

where the threshold  value is the modulus of the angle, which is between 0° and 180°.

For checking BBFs occurrence, the phase angle variations $\Delta \theta_{1}\;$ and $\Delta \theta_{2}$ which represent the total variation of $\;\theta_{1}^{\prime \prime }$, $\theta_{2}^{\prime \prime }$ respectively resulting from the combined magnitude and internal phase angle variation are calculated directly from the complex spectrum obtained from FFT of the new case (new) stator current:

(16) $$\Delta \theta_{1}=\theta_{1r}^{\prime \prime }-\theta_{1new}^{\prime \prime }\;\Delta \theta_{2}=\theta_{2r}^{\prime \prime }-\theta_{2new}^{\prime \prime }+\Delta \varphi \;diff=\Delta \theta_{1}+\Delta \theta_{2}$$

where diff is the modulus of the angle, which is between 0° and 180°.

If the condition diff≤threshold is satisfied, the motor is confirmed at healthy condition otherwise a broken bar fault is detected.

This adaptive threshold determination procedure is summarized in Section 5.3. 

## 4. Analyzing the Variation of Main Sideband Phase Angle under Different Conditions

In this section and as introduced in the previous section, the effect of inertia change, load variation, and BBFs on the main sideband components $i_{1}^{\prime \prime }$ and $i_{2}^{\prime \prime }$ (calculated using Equations (9) and (10)) will be studied and investigated graphically supported with many simulation cases.

### 4.1. Main Sideband Phase Angle Variation under Healthy Conditions

#### 4.1.1. Under Inertia Changing

**Figure 3:**
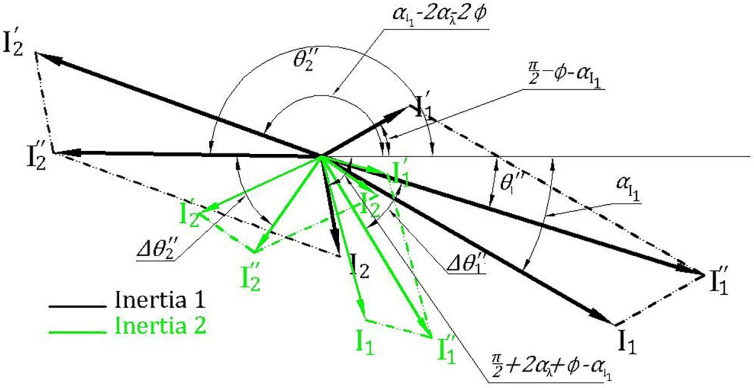
A phasor diagram representing the impact of inertia changes on $i_{1}^{\prime \prime }\;\mathrm{and}$ $i_{2}^{\prime \prime }$ current components under healthy conditions.

In the case of inertia variation from inertia 1 (black) to higher inertia 2 (green), parameters $\alpha_{I_{1}}$ and β change, but the values of $\phi ,\;i_{1}^{ο}$ remain constant as shown in Figure 3. The change in $\alpha_{I_{1}}$ results in reducing the angle $\theta_{1}^{\prime \prime }$ by the same amount of increment in $\;\theta_{2}^{\prime \prime }$, whereas the change in β causes changes to the magnitudes of $\;i_{1}^{\prime \prime },\;i_{2}^{\prime \prime }$, and their angles $\;\theta_{1}^{\prime \prime }$,$\;\theta_{2}^{\prime \prime }$.

**Figure 4:**
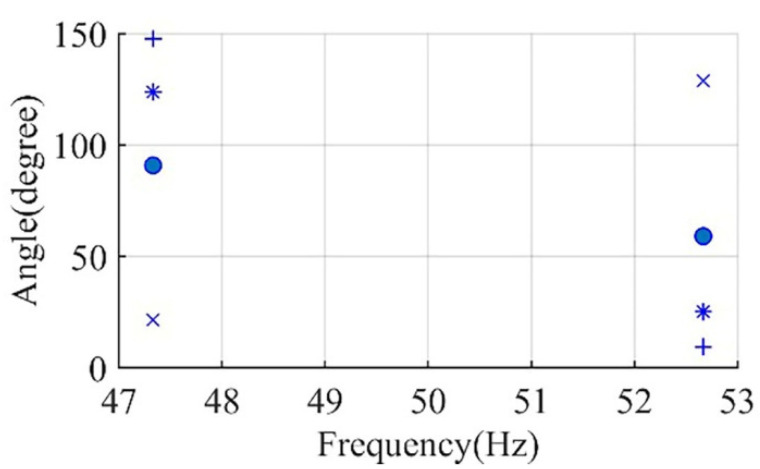
Motor I phase angle of left sideband and right sideband components for healthy conditions at inertia of; inertia of 0.063 kg·m^2^ (*), inertia of 0.63 kg·m^2^ (●), inertia of 0.93 kg·m^2^ (×), and inertia of 5.7 kg·m^2^ (+).

**Figure 5:**
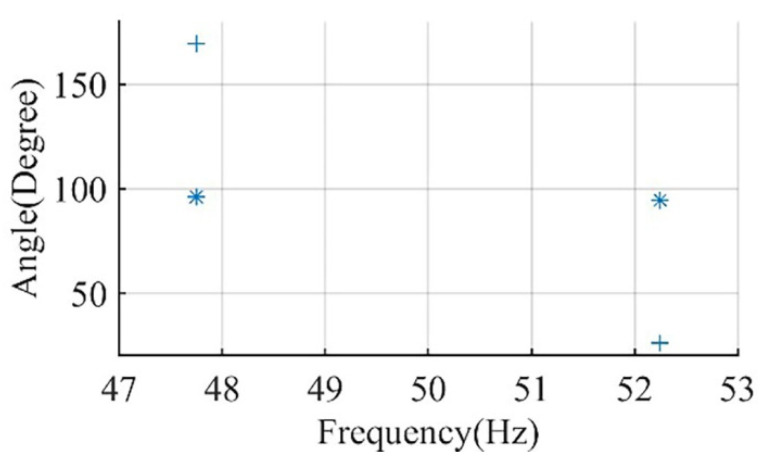
Motor II phase angle of left sideband and right sideband components for healthy conditions at; inertia of 16.84 kg·m^2^ (+), and inertia of 0.041 kg·m^2^ (*).

In the simulation studies for Motor I and Motor II, the inertia is varied from motor inertia (0.063 and 0.041 kg·m^2^, respectively) to the maximum inertia that each motor can accelerate according to NEMA MG-1 (5.7 and 16.84 kg·m^2^, respectively). The effect of this variation on the phase angle of the main sideband components is recorded in Figure 4 and Figure 5 for Motor I and Motor II, respectively.

For example, in Figure 4, the change in the inertia of Motor I from 0.063 to 0.93 kg·m^2^ affects the phase angle of the left main sideband component to change from 123.8° to 21.6° $\left(∆\theta_{1}^{\prime \prime }=102.2^{{}^{\circ}}\right)$ and the phase angle of the right main sideband component to change from 25.2° to 128.82° $\left(∆\theta_{2}^{\prime \prime }=-103.6^{{}^{\circ}}\right).$ Thus, the increase in $\theta_{1}^{\prime \prime }$ is approximately equal to the reduction in $\theta_{2}^{\prime \prime }$, which matches with the representation of current components in Figure 3 and the derivation in the previous section. For any healthy condition, the variation of main sideband phase angle will always be small.

#### 4.1.2. Under Load Changing

**Figure 6:**
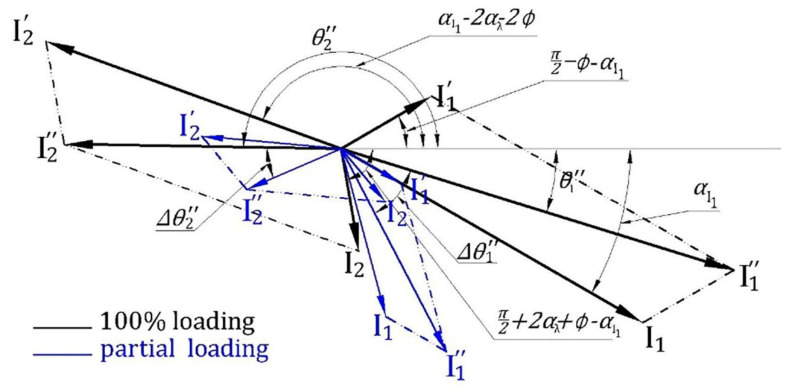
A phasor diagram representing the impact of load variation (from full loading to partial loading) on $i_{1}^{\prime \prime }\;and$ $i_{2}^{\prime \prime }$ current components under healthy conditions.

In healthy conditions, when the motor load changes from load 1 (black) to load 2 (blue), parameters $\alpha_{I_{1}},\;\beta ,\;\phi \;$ and $i_{1}^{ο}$ change as illustrated in Figure 6. The effect of the change in both $\alpha_{I_{1}}\mathrm{and}\;\beta$ is similar to that in the case of inertia variation. However, $i_{1}^{ο}$ changes because it is dependent on rotor backward flux, which is related to stator flux and hence stator current varies by a linear relationship as long as the motor is considered to operate in the linear zone of the magnetizing curve [4].

In other words, $i_{1}^{ο}$ changes approximately with the same rate of the change in the stator current. Although it has an equal effect on all current components, the change in ϕ makes $∆\theta_{2}^{\prime \prime }$ deviates from $∆\theta_{1}^{\prime \prime }$ by an amount equal to  ∆ϕ. Thus, it can be mentioned that the difference between $∆\theta_{2}^{\prime \prime }$ and $∆\theta_{1}^{\prime \prime }$ in case of changing loading condition results from the variations of  ϕ and β.

**Figure 7:**
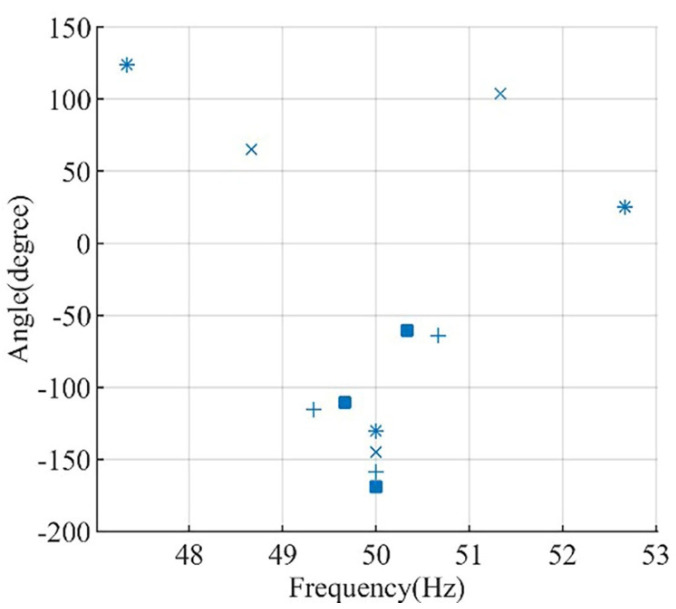
Motor I phase angle of left sideband, right sideband and fundamental components for healthy conditions at; 10% loading (■), 25% (+), 50% loading (×), and full loading (*).

To analyze the effect of variable loading on the phase angle of the main sideband components, Motor I is simulated under different loading conditions ranging from 10% to 100% of the rated motor load, and results are revealed in Figure 7. When ∆ϕ is added to $\;∆\theta_{2}^{\prime \prime }$, the result is approximately equal to $\;∆\theta_{1}^{\prime \prime }$, and typically the difference is below 10° in all simulated tested cases.

For example, the loading change in Motor I from full load to half load causes the phase angle of the left main sideband component to change from 123.8° to 64.9° $\left(∆\theta_{1}^{\prime \prime }=58.9^{{}^{\circ}}\right)$, while the phase angle of the right main sideband component changes from 25.2º to 103.7 $\left(∆\theta_{2}^{\prime \prime }=-78.5^{{}^{\circ}}\right)$ and $∆\phi =15^{{}^{\circ}}$. Clearly, the increase in $\theta_{1}^{\prime \prime }$ is approximately equal to the decrease in $\theta_{2}^{\prime \prime }$ plus the increase in the power factor angle ϕ, which is the evidence that in any case represents the healthy condition, the variation of the main sideband phase angle is small. The change in the location of the main sideband frequency components in the frequency domain is due to the change in motor slip with load variation.

### 4.2. Main Sideband Phase Angle Variation under Fault Conditions

**Figure 8:**
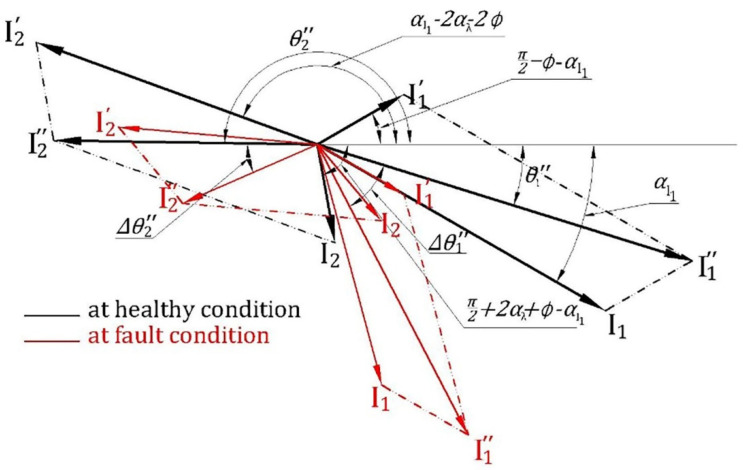
A phasor diagram representing the impact of BBF on $i_{1}^{\prime \prime }\;and$ $i_{2}^{\prime \prime }$ current components.

When the motor status changes from the healthy case (black) to a broken bar fault (red), parameters $\alpha_{I_{1}},\;\alpha_{\lambda }\;$and $i_{1}^{ο}$ change as displayed in Figure 8. The effect of change in $\alpha_{I_{1}}\;and\;i_{1}^{ο}$ is the same as that in the case of inertia variation whereas, the change in $\alpha_{\lambda }\;$makes $∆\theta_{2}^{\prime \prime }$ deviate from $∆\;\theta_{1}^{\prime \prime }$ by an amount equal to twice the change in$\;\alpha_{\lambda }$.

**Figure 9:**
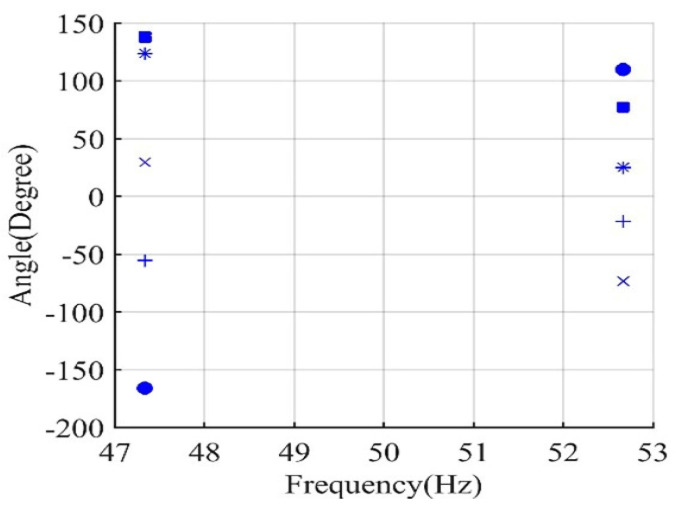
Motor I vector representation of left sideband and right sideband components for; adjacent 2 BBR at inertia 0.63 kg·m^2^ (●), non-adjacent 2 BBR at inertia 0.63 kg·m^2^ (×), BBR at inertia 0.63 kg·m^2^ (■), HBBR at 5.7 kg·m^2^ (+), and healthy conditions at 0.063 kg·m^2^ (*).

Motor I is simulated with a half-broken bar (HBBR), one broken bar (BBR), adjacent two broken bars (adjacent 2 BBR), and non-adjacent two broken bar separated by pole pitch with different inertia to analyze the effect of BBF on the phase angle of the main sideband component. Magnitudes and angles of $i_{1}^{\prime \prime }$ and$\;i_{2}^{\prime \prime }$ are recorded as shown in Figure 9. It is found that the difference between $∆\theta_{1}^{\prime \prime },\;∆\theta_{2}^{\prime \prime }$ in case of BBFs is ranging from 29° for HBBR at 25% loading, and 0.063 kg·m^2^ to 170° and 140° for non-adjacent 2 BBR at pole pitch distance and full load at 0.63 and 0.063 kg·m^2^, respectively, as will be illustrated in the results section.

**Figure 10:**
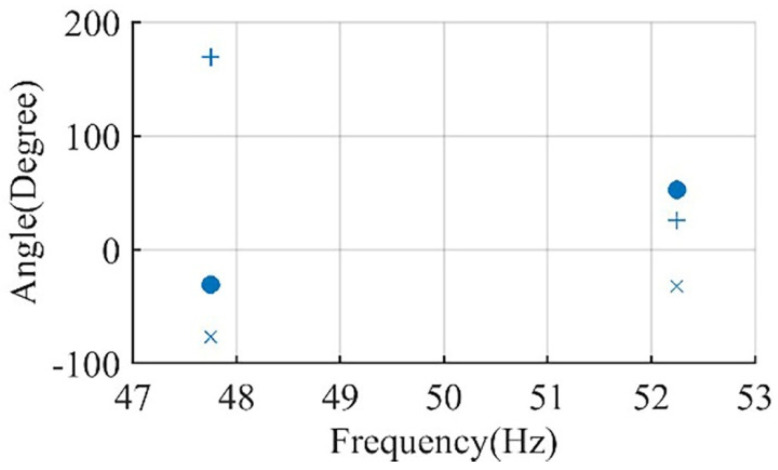
Motor II vector representation of left sideband and right sideband components for; inertia of 16.84 kg·m^2^ (+), HBBR at 16.84 kg·m^2^ (×), and HBBR at 0.042 kg·m^2^ (∙).

In addition, for Motor II, HBBR with different inertia is simulated to confirm the relation between $∆\theta_{1}^{\prime \prime },\;∆\theta_{2}^{\prime \prime }$ for BBFs, and the results are displayed later in Figure 10.

## 5. Implementation of the Proposed Scheme

For the generalization of the proposed method to be suitable for an induction motor, the implementation of the proposed scheme that does not need any setting is presented in this section. Four stages are implemented within the proposed technique. Namely:

▪ Data acquisition stage: It includes the sampling process of the current and voltage signal and storing samples to reach the required frequency resolutions.
▪ Data processing stage: It includes data windowing block, FFT block, and localization of the main sideband component block to obtain the magnitude and angle of these sideband components.
▪ Adaptive threshold determination and fault detection stage: It includes a method for adaptive threshold calculation to differentiate between healthy and BBFs conditions.
▪ Severity index calculation stage: It provides a severity index to designate the severity of the fault.

Each stage is described in detail in the following subsections.

### 5.1. Data Acquisition Stage

In this stage, the steady-state single-phase stator current received from the current sensor is sampled at a 12 kHz sampling rate, and the current samples are stored until reaching the minimum length of data required to differentiate between main sideband components and power frequency component that is only achieved if $f_{ο}\leq 2s$ where $\;f_{ο}\;$ is the frequency resolution, $\;f_{ο}=\frac{sampling\;frequency\;\left(f_{s}\right)}{number\;of\;samples\;\left(N\right)}$ [32].

The frequency resolution should be adequately selected such that the main sideband components can be distinguished at the lighter load at which the motor can operate. However, increasing the frequency resolution exaggeratedly will result in increasing data length and hence a larger memory is required and a longer period is needed to start diagnosing process. After reaching the minimum data length required, the scheme receives new samples of one complete cycle and removes old samples keeping the length of data fixed.

In addition, the steady-state voltage signal received from voltage sensor is sampled to be used for calculating the power factor angle in the case of variable loading conditions.

### 5.2. Data Processing Stage 

In this stage, the sampled signal is windowed to improve the accuracy of the data obtained from FFT then the sideband components are localized in the frequency spectrum. This stage can be divided into three main blocks as follows:

#### 5.2.1. Signal Windowing Block

The sampled signals are truncated using a window of length that satisfies the condition required to distinguish power and sideband components. Hann window is selected as a data window because of its accuracy in phase estimation compared to other data windows [33]. The Hann window can be expressed by:

(17) $$w\left(n\right)=0.5\left(1-cos\left(2\pi \frac{n}{N}\right)\right),\;\;\;\;\;0\leq n\leq N$$

#### 5.2.2. Fast Fourier Transformer Block

Using this block, the magnitude and phase angle of each frequency component in the signals received from the previous block is calculated using the FFT algorithm.

However, FFT is used for signal frequency analysis in this paper for its simplicity; any frequency analysis method that provides accurate frequency, amplitude, and phase angle estimation can be used.

#### 5.2.3. Localization of Main Side Band Components

The current and voltage data received from the previous block are in form of two arrays. One contains complex coefficients of discrete Fourier transform for the windowed signal using FFT algorithm where the array magnitude is $\left[m\right],\;\left[m_{v}\right]\;$ and its phase angle is $\left[\theta \right],\;\left[\theta_{v}\right]$, while the other array contains component frequencies $\;\left[freq\right],\left[freq_{v}\right]\;$ for current and voltage signal respectively. The algorithm for main sideband components localization can be summarized in the following five steps:

Step 1: Determine the current power frequency components that have the maximum magnitude $\left(m_{f}\;\right)$ and its associated frequency $\;\left(f\;\right)\;$ from the two arrays, $m_{f}=\max\left[m\right]$ and $f=freq\left(m_{f}\right)$.
Step 2: Determine the frequency searching zone for the main side band components, which is limited by $\left[\left(1-4s_{max}\right)f\right]\;$ and $\left[\left(1+4s_{max}\right)f\right]\;$ where $s_{max}$ is the maximum slip at which the motor can operate and its associated magnitude $\left[m_{\left(1-4s_{max}\right)f},\;\cdots ,\;m_{\left(1+4s_{max}\right)f}\;\right]$.
Step 3: Search for the local maxima in the magnitudes of the searching zone such that $\;m_{i-1}\leq m_{i}\leq m_{i+1}\;$ and then arrange them in descending order in$\;\left[m_{l}\right]\;$and their associated frequencies in $\;\left[f_{l}\right]$.
Step 4: Ensure that the frequency of the first $f_{l}\left(m_{l}\left(1\right)\right)\;$ and the second greatest local maxima $f_{l}\left(m_{l}\left(j+1\right)\right),\;j=1,2,\ldots$ is symmetrical around the power frequency otherwise; take the third greatest local maxima instead of the second one and check for this condition.
Step 5: When the condition in Step 4 is fulfilled, set the following:
  (18) $$\;I_{new}=m_{f},\;\;\;\phi_{new}=\theta_{v}\left(f\right)-\theta \left(f\right)$$
  (19) $$f_{I_{1new}^{\prime \prime }}=\min\left(f_{l}\left(m_{l}\left(1\right)\right),f_{l}\left(m_{l}\left(j+1\right)\right)\right)$$
  (20) $$I_{1new}^{\prime \prime }=m(f_{I_{1new}^{\prime \prime }}),\;\;\;\theta_{1new}^{\prime \prime }=\theta \left(f_{I_{1new}^{\prime \prime }}\right)$$
  (21) $$f_{I_{2new}^{\prime \prime }}=\max\left(f_{l}\left(m_{l}\left(1\right)\right),f_{l}\left(m_{l}\left(j+1\right)\right)\right)$$
  (22) $$I_{2new}^{\prime \prime }=m(f_{I_{2new}^{\prime \prime }}),\;\;\;\theta_{2new}^{\prime \prime }=\theta \left(f_{I_{2new}^{\prime \prime }}\right)$$

### 5.3. Adaptive Threshold Determination and Fault Detection Stage

As mentioned before, one of the distinguishing features of the proposed scheme is to calculate adaptively the threshold value to distinguish between healthy and faulty cases. This section uses the same terminology as the rest of the paper, again the subscripts: r, new, m, and mnew indicate for reference case (r), any new case (new), the model (m) in Figure 2 and finally the new case current components calculated using this model (mnew) suggested in this paper, respectively. 

Adaptive threshold determination can be summarized in the following steps:

1. Calculate $I_{r},I_{1r}^{\prime \prime },,I_{2r}^{\prime \prime },\;\theta_{1r}^{\prime \prime }\;,\;\theta_{2r}^{\prime \prime }\;and\;\varphi_{r}$ at a reference healthy condition for the motor to be monitored using FFT at any inertia and under any loading conditions.
2. Calculate the reference values $\beta_{r}$ and $I_{1}^{ο}{}_{r}$ from Equations (12) and (14). Only positive and real values of them will be accepted.
3. The previous two steps are carried out once for the healthy motor in the commissioning phase.
4. Calculate $I_{new},I_{1new}^{\prime \prime },I_{2new}^{\prime \prime },\;\theta_{1new}^{\prime \prime }\;,\;\theta_{2new}^{\prime \prime }\;and\;\varphi_{new}$ for the new current samples using FFT.
5. Calculate the value of $\beta_{new}$ from Equations (12) and (14). Using $\;I_{1}^{ο}{}_{r}$, which has been obtained from Step (2) to limit the variation to be in $\beta_{new}$ only. The value of $\beta_{new}$ that has the largest deviation from $\beta_{r}$ will be selected to provide a safety margin to avoid false diagnosis.
6. Calculate the magnitudes of $i_{1\mathrm{mnew}}^{\prime \prime \prime },\;i_{1\mathrm{mnew}}^{\prime },\;i_{2\mathrm{mnew}}^{\prime }\;and\;i_{2\mathrm{mnew}}$ using Equations (7), (5), (8) and (6), respectively, with the values of $\;\beta_{new}$ and $i_{1}^{ο}{}_{r}$
7. To study the magnitude variation effect of current components calculated in Step (5) on $\;\theta_{1}^{\prime \prime }\;,\;\theta_{2}^{\prime \prime }$, the model introduced in Figure 2 is used to calculate $\;\theta_{1\mathrm{mnew}}^{\prime \prime }\;,\;\theta_{2\mathrm{mnew}}^{\prime \prime }$ using the data obtained from Step (5).
8. Calculate threshold value using Equation (15).
9. For checking BBFs occurrence, calculate the angle difference using Equation (16).
10. If the condition diff≤threshold is satisfied, the healthy condition is confirmed otherwise a broken bar fault is detected.

### 5.4. Severity Index Calculation Stage

If the previous stage has indicated that there is a BBF, an index is needed to assess the severity of the fault. Thus, in this subsection, a method is introduced to indicate the severity of the fault that is largely independent of load inertia and loading conditions. The proposed method can be summarized in the following steps:

1. Calculate the values of $i_{1}^{ο}{}_{\mathrm{mnew}}$ from Equations (12) and (14) using $\beta_{r}$ value, which has been obtained from Step (2) in the threshold determination module.
2. Calculate the average of $i_{1}^{ο}{}_{\mathrm{mnew}}$ values.
3. Calculate the corrected current as follows, as $\;i_{1}^{ο}{}_{\mathrm{mnew}}$ is directly proportional to $\;I_{new}$:
  (23) $$i_{1}^{ο}{}_{mcorrected}=i_{1}^{ο}{}_{\mathrm{mnew}}\times \frac{I_{r}}{I_{new}}$$
4. Calculate the severity index:
  (24) $$severity\;index=\frac{i_{1}^{ο}{}_{mcorrected}\;}{I_{1}^{ο}{}_{r}}$$

**Figure 11:**
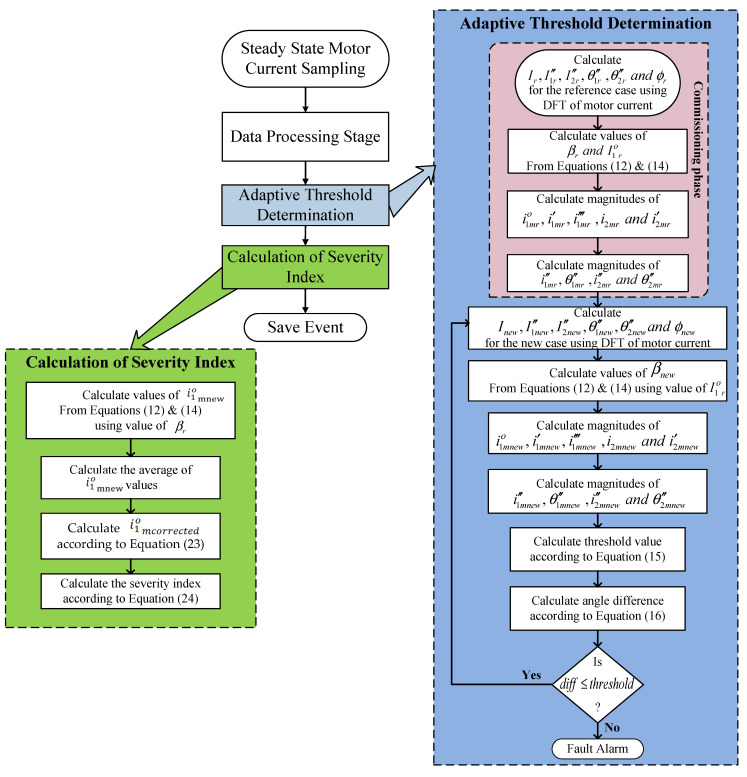
A flowchart summarizing the overall proposed scheme.

A flowchart summarizing the overall proposed scheme is presented in Figure 11.

## 6. Testing Results for Proposed Scheme Performance 

The proposed adaptive scheme has been validated using two simulated induction motor models (Motor I and Motor II previously described in Table 1), in addition to the experimental dataset provided online in [34]. The threshold has been determined using the proposed procedure steps in Section 5. The proposed scheme has been extensively tested against system inertia variation, variable loading conditions, noisy environment, and various BBFs severity as will be shown in the next subsections. 

### 6.1. Under Different System Inertia

As discussed in [6], inertia has a dominant effect on the main sideband magnitudes and their angles. In this paper, each simulated motor model has been tested against a wide range of inertia ranging from motor inertia and the maximum inertia that the motor can accelerate. 

For simulated Motor I, Cases 1, 4 and 8 in Table 2 illustrate the effect of inertia variation (5.7, 0.63, and 0.925 kg·m^2^) on both measured main sideband phase angles and adaptive threshold estimation under healthy conditions at full load conditions. The obtained results in the table confirmed such cases as healthy cases since the calculated difference is less than the adaptive estimated threshold.

Conversely, the results for fault conditions (Cases 2, 6 and 9 in Table 2) ensure the accurate performance of the proposed scheme in detecting BBFs occurring during any inertia value (5.7, 0.63, and 0.925 kg·m^2^). As obviously shown, different faults of BBR, 2 BBR and 3 BBR are accurately detected. 

The same performance is achieved when testing Motor II, as illustrated in the simulation results presented in Table 3, where half BBR is accurately detected in both Cases 2 and 3, although the inertia is different. Cases 1 and 2 prove the ability of the proposed scheme to successfully monitor the motor conditions and adaptively change the threshold. For example, the threshold calculated using the proposed scheme has been changed adaptively from 64.08° at the healthy condition to 48.49° at fault condition (Cases 1 and 2 in Table 3) to be able to detect half BBR, although it is a less severe fault. 

### 6.2. Under Different Loading Conditions 

To further investigate the proposed scheme capability, the effects of variable loading on both measured main sideband phase angles and estimating threshold under healthy and faulty conditions are studied. Thus, Motor I is widely tested under variable loading conditions from 100% to 10% of rated load which is equivalent to slip variation from 0.029 to 0.0026. Table 2 introduces sample of these testes at 10% (Cases 16, 17 and 18), 25% (Cases 13, 14, 15 and 21), 50% (Cases 11 and 12) and 100% (Cases 1 to 10 and 19 and 20). As expected, the proposed scheme is able to differentiate perfectly between healthy and faulty conditions at all tested loading conditions and even detects incipient fault at 10% loading condition in contrary to methods suggested in [35,36] that can detect full broken bar at 25% loading.

Although the methods introduced in [37] can detect full broken bar at no-load, the method in [38] requires a long acquisition time (about 100 s), and the method in [37] was tested on no load condition at slip *s* = 0.005. Conversely, the proposed scheme can detect BBFs at *s* = 0.0026 (10% loading condition), which means that the proposed scheme can detect more difficult faults than introduced in [37]. 

The windows with an acquisition time of 12, 9, and 3 s give frequency resolution of 0.083, 0.11, and 0.33 Hz are used in this paper for 10%, 25%, and above 50% loading condition, respectively. 

### 6.3. Under Different Fault Severity 

As discussed before that a non-adjacent broken bar fault is one of the most common misdiagnosed faults for several reported methods. On contrary, the proposed scheme is designed to detect all different levels of fault severity, either faults that are easy to be detected such as full broken bar, adjacent 2 BBR, and 3 adjacent BBR, or faults that are difficult to detect, such as half BBR and non-adjacent 2 BBR at pole pitch distance. To achieve that, the proposed scheme is designed without any need for special flux sensors or the need for a three-phase current sensor; it is designed to detect effectively incipient BBFs at 10% loading condition using single-phase current and voltage sensors.

Comprehensive tests have been carried out to evaluate the effect of fault severity on both measured main sideband phase angles and estimate the threshold under faulty conditions. The simulation results of Cases 5 and 10 in Table 2 have ensured that the proposed scheme has detected correctly non-adjacent 2 BBR for Motor I at 0.625 and 0.0625 kg·m^2^, respectively. In addition, half BBR faults are detected at full loading, 50%, 25% and 10% loading as illustrated from Cases 3, 12, 15 and 18, respectively, as the measured differences were higher than the estimated adaptive threshold in such cases.

Table 3 shows the performance evaluation for the proposed scheme on Motor II for a limited number of cases to just confirm that the proposed scheme is able to differentiate between healthy motor and motor with BBFs, regardless of the motor characteristics and design. A different fault location has been tested, and it was found that the proposed scheme can detect BBFs regardless of its fault location.

### 6.4. In a Noisy Environment

To examine the effectiveness of the proposed scheme in a noisy environment, white noise is injected into the motor current signal such that the signal to noise ratio (SNR) is equal to 20 and 18.5 dB at full loading and 25% loading, respectively, for Motor I. 

Consequently, Cases 19 and 20 in Table 2 illustrate the correct diagnosis of the proposed scheme at full loading under the presence of noise as the threshold has been changed adaptively from 21.2° at the healthy condition to 36.9° at faulty conditions to be able to detect half BBR as a less severe fault. Meanwhile, Case 21 ensures the capability of the proposed scheme in detecting incipient faults under light loading conditions (25%) and noisy environments. 

### 6.5. Faulty Severity Determination 

**Figure 12:**
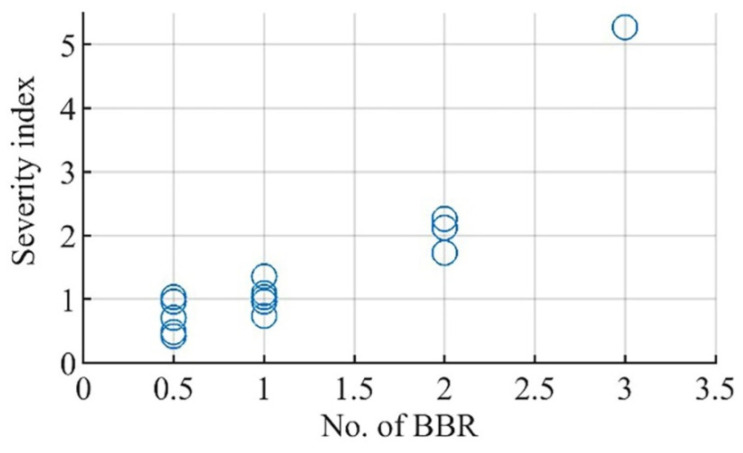
Severity index of Motor I against number of broken bars.

The last column in Table 2 and Figure 12 demonstrate the severity index calculations for simulated BBFs in Motor I. As shown, the proposed index shows a good correlation with the actual fault severity; for example, the values of severity index were estimated by 0.965, 1.034, 1.36, and 1.09 for BBR at full load, 50% and 25% as illustrated in Cases 2, 7, 11 and 14. Besides, using this index, the severity of non-adjacent 2 BBR at pole pitch distance, which is commonly misestimated, can be correctly estimated as a two-bar fault by severity index of 1.73 and 2.26 in Cases 5 and 10, respectively.

Conversely, with the change in loading conditions, the severity index is over-estimated for some less severe faults of half BBR. As shown, the index is overestimated by 1.030 and 0.96 in Cases 12 and 15 under partial loading of 50% and 25%, respectively.

### 6.6. Validation of the Proposed Scheme Using Real Experimental Dataset

The proposed scheme has also been validated under different severity conditions using the experimental dataset provided online in [34]. The dataset is utilized in several published work such as [39,40].

**Figure 13:**
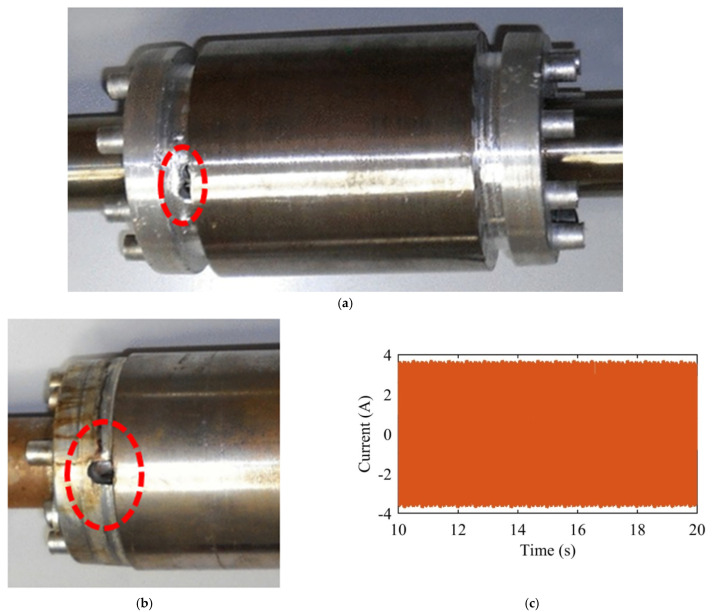
Sample of rotor photos and current waveforms for the dataset provided in [34]. (**a**) A photo of the rotor with two holes drilled at one bar [39]. (**b**) A photo of the rotor with one broken bar [40]. (**c**) The current waveform of one broken bar case.

The test stand was composed from a low voltage induction motor coupled to a dc machine has inertia 0.11 kg·m^2^. The motor has the following characteristics: 400 V, 1.5 kW, 2-poles, 3.25 A, and 2860 rpm at full load. The phase current was captured by a current transformer and sampled with 5 kHz. Bars were drilled to represent a partial broken bar and one broken bar. The dataset has contained the following cases at full load and constant inertia:

- Healthy;
- One bar with 3 mm diameter hole;
- One bar with two 3 mm diameter holes each;
- One bar with two 4 mm diameter holes each (as illustrated in Figure 13a);
- One broken bar (as illustrated in Figure 13b while the current waveform of such case is shown in Figure 13c).

By applying the proposed scheme for the aforementioned cases, the obtained results are illustrated in tabulated format in Table 4. The achieved results demonstrated the perfect ability of the proposed scheme to detect different broken bar faults under different severity conditions including incipient faults. Moreover, the proposed scheme provided a robust index that is highly sensitive to fault severity.

## 7. Conclusions

This paper presented an analytical approach for investigating the variation of the magnitude and phase angle of the main sideband components under different load inertia, variable load, and fault conditions. Furthermore, a geometrical representation is developed to describe the effects of contrasting operating conditions on the phase angle of the main sideband frequency components. Extensive simulations are applied to validate this analytical approach. A novel scheme to detect BBFs is developed based on this approach. This scheme provides an automatic adaptive threshold to discriminate between the healthy and the faulty cases without the need for any settings. This scheme was validated against variable loading conditions from 100% to 10% of the rated load, a wide range of inertia, and different BBFs severities. Moreover, the performance of this scheme is tested in the case of a noisy environment. Finally, this scheme was tested using experimental dataset, including incipient BBFs. The results showed that the proposed scheme could effectively detect all BBFs that include partial, HBBR, and non-adjacent BBR under all inertia, loading conditions, and in a noisy environment. In addition, the results showed that this scheme is independent of the motor parameters.

## Figures and Tables

**Table 1:** Electrical data of the two tested motors.

Data	Motor I [4]	Motor II
Power	11 kW	11 kW
Voltage (rms)	380 V	380 V
No. of poles	4	6
Rated slip	2.9%	2.3%
Number of stator slots	36	72
Number of rotor bars	28	58
Number of turns	27	6
Silicon steel material	M19_29G	M19_24G

**Table 2:** Testing the proposed scheme on Motor I.

Case No.	Case Description			Performance of the Proposed Scheme			
	Healthy/BBFs	Loading Condition (%)	Inertial Condition (kg·m^2^)	Estimated Threshold (°)	Calculated Phase Angle Difference (°)	Status	Severity Index
1	Healthy	Full load	5.7	49.97	8.62	Healthy	--------
2	BBR	Full load	5.7	52.09	120	Fault	0.965
3	Half BBR	Full load	5.7	56.28	132.5	Fault	0.418
4	Healthy	Full load	0.625	22.58	0.91	Healthy	--------
5	Non-adjacent 2 BBR	Full load	0.625	20.72	170.1	Fault	1.73
6	2 BBR	Full load	0.625	23.27	155.1	Fault	2.126
7	BBR	Full load	0.625	23.74	145.30	Fault	1.034
8	Healthy	Full load	0.925	23.59	1.43	Healthy	--------
9	3 BBR	Full load	0.925	19.2085	104.5312	Fault	5.27
10	Non-adjacent 2 BBR	Full load	0.0625	19.99	141.4	Fault	2.26
11	BBR	50%	0.0625	22.23	60.9	Fault	1.36
12	Half BBR	50%	0.0625	24.56	43.44	Fault	1.030
13	Healthy	25%	0.0625	26.42	2.72	Healthy	--------
14	BBR	25%	0.0625	24.16	50.6	Fault	1.09
15	Half BBR	25%	0.0625	26.09	28.94	Fault	0.96
16	Healthy	10%	0.0625	29.00	0.71	Healthy	--------
17	BBR	10%	0.0625	26.19	154.6	Fault	0.736
18	Half BBR	10%	0.0625	27.81	102.38	Fault	0.708
19	Healthy + 20 db Noise	Full load	0.0625	21.2	9.75	Healthy	--------
20	Half BBR + 20 db Noise	Full load	5.7	36.9	76.03	Fault	0.484
21	Half BBR + 18.5 db Noise	25%	0.0625	20.67	34.74	Fault	1.96

**Table 3:** Testing the proposed scheme on Motor II.

Case No.	Case Description			Performance of the Proposed Scheme		
	Healthy/BBFs	Loading Condition (%)	Inertial Condition (kg·m^2^)	Estimated Threshold (°)	Calculated Difference (°)	Status
1	Healthy	Full load	0.0410	64.08	5.85	Healthy
2	Half BBR	Full load	0.0410	48.49	169.67	Fault
3	Half BBR	Full load	16.84	48.56	60.58	Fault
4	Healthy	90% loading	0.0410	49.57	3.13	Healthy

**Table 4:** Testing the proposed scheme using the experimental dataset.

Case No.	Case Description			Performance of the Proposed Scheme			
	Healthy/BBFs	Loading Condition (%)	Inertial Condition (kg·m^2^)	Estimated Threshold (°)	Calculated Phase Angle Difference (°)	Status	Severity Index
1	Healthy	Full load	0.11	13.015	3.2	Healthy	--------
2	Healthy	Full load	0.11	14.5	3.4	Healthy	--------
3	A bar with one 3 mm diameter hole	Full load	0.11	18.5	77.94	Fault	0.78
4	One bar with two 3 mm diameter holes each	Full load	0.11	18.069	93.836	Fault	4.17
5	One bar with two 4 mm diameter holes each	Full load	0.11	18.071	160.78	Fault	5.02
6	One full broken bar	Full load	0.11	13.5	166.49	Fault	7.2

## Data Availability

Not applicable.

## Nomenclature

**Symbol**	**Definition**
AI	Artificial Intelligence
BBF	Broken Bar Fault
BBR	Broken Bar
EMD	Empirical Mode Decomposition
EMF	Electromotive Forces
ESPRIT	Estimation of Signal Parameters via Rotational Invariance Techniques
FC	Fundamental Current Component
FEM	Finite Element Method
FFT	Fast Fourier Transform
HBBR	Half-Broken Bar
HHT	Hilbert–Huang transform
MCSA	Motor Current Signature Analysis
MUSIC	Multiple Signal Classification
NEMA	National Electrical Manufacturers Association
RLSH	Left-Side Harmonic
RMS	Root Mean Square
RSH	Right Sideband Harmonic
SNR	Signal To Noise Ratio
WT	Wavelet Transform
$E_{1-2s}$ and $E_{1+2s}$	The RMS of electromotive force of the LSH and RSH respectively
$e_{1-2s}$ $\;\mathrm{and}\;e_{1+2s}$	The instantaneous of electromotive force of the LSH and RSH respectively
f	The supply frequency
$f_{r}$	Rotor frequency component
$I_{a}$	The stator current
J	System inertia
$I,I_{l}$ and $I_{r}$	The RMS of fundamental current, LSH and RSH components respectively
$i_{1}^{\prime },\;i_{2},\;\;i_{1}^{\prime \prime \prime },\;\;i_{2}^{\prime },\;\;i_{2}^{\prime \prime }$ and $i_{1}^{\prime \prime }$	Notations for different current components, where subscript (1) and (2) indicate components related to LSH and RSH respectively
$i_{1}^{ο}$	Initial left sideband current
$i_{1}^{ο}{}_{mcorrected}$	The corrected values of $i_{1}^{ο}$
P	Number of poles
s	Motor slip
ω	Synchronous speed
$Z_{1-2s}$ and $Z_{1+2s}$	The circuit impedance for LSH and RSH respectively
Z and ϕ	The magnitude and the angle of the equivalent circuit impedance at supply frequency
$\alpha ,\;\alpha_{l}$ and $\alpha_{r}$	The phase angles of FC, LSH, and RSH
$\alpha_{\lambda }$	The angle of the fundamental flux linkage
β	A parameter equals $\frac{3{P\lambda }^{2}}{8Js\omega \left|Z\right|}$
$\;\theta_{1}^{\prime \prime }$ and $\theta_{2}^{\prime \prime }$	The phase angle of the current components $i_{1}^{\prime \prime }\mathrm{and}\;i_{2}^{\prime \prime }$, respectively
Δθ	The change in the angle θ
λ	The RMS value of the fundamental flux linkage
$\lambda_{fund}$	The instantaneous value of the fundamental flux linkage
$\ldots_{r}$	The subscript$\;\left(r\right)$ denotes reference cases
$\ldots_{new}$	The subscript$\;\left(new\right)$ denotes new cases
$\ldots_{\mathrm{mnew}}$	$\mathrm{The}\;\mathrm{subscript}\;\left(\mathrm{mnew}\right)$ indicates the new case current components calculated using the model in Figure 2
$\ldots_{m}$	The subscript (*m*) indicates the model indicates the model
